# Chronic Sleep Deprivation Altered the Expression of Memory-Related Genes and Caused Cognitive Memory Dysfunction in Mice

**DOI:** 10.3390/ijms252111634

**Published:** 2024-10-29

**Authors:** Xiang Wang, Hanqing Chen, Tian Tang, Xiang Zhan, Shu Qin, Taijun Hang, Min Song

**Affiliations:** Department of Pharmaceutical Analysis, China Pharmaceutical University, Nanjing 211100, China; wangxx0626@163.com (X.W.); 15617515335@163.com (H.C.); tt13739257904@163.com (T.T.); 18756935995@sina.cn (X.Z.); qsqinshu@163.com (S.Q.)

**Keywords:** chronic sleep deprivation, RNA-seq, sporadic Alzheimer’s disease, differential gene expression, calcium signaling

## Abstract

Lack of sleep, whether acute or chronic, is quite common and negatively affects an individual’s memory and cognitive function. The question of whether chronic sleep deprivation (CSD) causes cognitive impairment to arise and progress is not well studied. To investigate the effects of CSD on memory and cognition, this study began by establishing a CSD mouse model. Behavioral experiments on animals revealed that CSD induced cognitive behavioral abnormalities reminiscent of Alzheimer’s disease. Western blot experiments further demonstrated a considerable increase in amyloid-β (Aβ) expression in the mouse brain following CSD. Meanwhile, the hub gene Prkcg was searched for in the cerebellum using RNA-seq and bioinformatics analysis. PKCγ (Prkcg) expression was significantly reduced, as demonstrated by RT-qPCR and Western blot validations. Additionally, CSD was associated with downregulated CREB expression, decreased expression of the endothelin-converting enzyme (ECE1), and increased phosphorylation of ERK1/2 downstream of PKCγ. These findings suggested that CSD down-regulated PKCγ expression, decreased ECE1 expression, impaired Aβ degradation, and affected the PKCγ/ERK/CREB pathway and the synthesis of memory-related proteins. Overall, this study highlighted how CSD modulated PKCγ-related metabolism, impacting Aβ clearance and the production of memory-related proteins. Such insights are crucial for understanding and preventing sporadic Alzheimer’s disease (sAD) associated with CSD.

## 1. Introduction

Numerous studies have highlighted a robust correlation between sleep deprivation and cognitive impairment. For instance, one study utilizing β-amyloid PET scans found that a single night of sleep loss in 20 healthy adults increased parenchymal β-amyloid burden by 5% [1]. Additionally, subsequent sleep on the second or third day failed to compensate for the impaired brain clearance observed in the sleep-deprived group [2]. Sleep was found to enhance the interstitial volume fraction by 60%, resulting in a twofold faster clearance of Aβ from the cerebral cortex [3]. Notably, severe sleep disorders have been linked with traumatic brain injury, which amplifies the burden of tau and Aβ proteins in the affected individual’s brain, thereby elevating the risk of developing AD [4]. These findings resonate with reports of sleep disruption occurring in the preclinical stages of AD. AD is characterized by the accumulation of Aβ and tau protein aggregates in vulnerable brain regions long before clinical dementia manifests [5].

Acute sleep deprivation has been shown to elevate soluble Aβ levels, increase the risk of Aβ plaque formation, and promote tau aggregation in the interstitial fluid (ISF) and cerebrospinal fluid (CSF) of mice in an AD animal model [6,7]. Chronic sleep deprivation in APP/PS1 mice over two months led to heightened senile plaque formation and increased production of amyloid-β1-42 in the cortex and hippocampus. Both APP/PS1 and WT mice exhibited significant neuronal apoptosis, neuronal mitochondrial damage, and activation of the caspase cascade in the hippocampal regions following prolonged sleep deprivation [8,9]. Moreover, chronic sleep deprivation resulted in abnormal expression of suprachiasmatic nuclear circadian rhythm genes in AD mice, elevated phosphorylation of tau231, and deposition of Aβ oligomers in the hippocampus and cortex [10]. Findings from human and genetically modified animal studies suggest that sleep deprivation is a chronic and enduring process, underscoring the importance of investigating how sleep loss contributes to cognitive impairment using the CSD model.

Traditionally, sleep research has focused on the interrelation of these states within neocortical and subcortical structures, neglecting cerebellar activity [11,12]. Recent research has uncovered that while the cerebellum’s somatosensory regions constitute a small portion of the brain, the default mode network and cognitive control functions occupy nearly half of the cerebellum [13]. Furthermore, contrary to previous beliefs about the cerebellum’s immunity to AD pathology, it is indeed vulnerable to Aβ toxicity, even in the early stages of the disease, with implications for cognition and memory function [13,14]. Studies of both familial and sporadic AD have identified the cerebellum as a susceptible region in affected patients, with higher concentrations of Aβ oligomers observed in cerebellar cortical areas [15,16,17]. Cerebellar atrophy is a characteristic feature of sAD, affecting connections within the default mode network [18,19]. Deficits in synaptic plasticity within the cerebellar cortex associated with motor learning disorders have been observed in AD mouse models [20]. Given the cerebellum’s intricate connections with the brain and the underlying neural networks governing sleep–wake regulation, it is worthwhile to explore how CSD impacts cerebellar changes and, consequently, cognitive memory function. In this study, wild-type 10-month-old mice underwent gentle sleep deprivation for six months, with 10 h of sleep deprivation per day. The effects of CSD were evaluated through behavioral tests, identification of characteristic proteins, screening of hub genes, and preliminary exploration of the underlying mechanisms.

## 2. Results

### 2.1. Chronic Sleep Deprivation Impaired Spatial Working Memory and Induced Anxiety-like Mood in Mice

**Figure 1:**
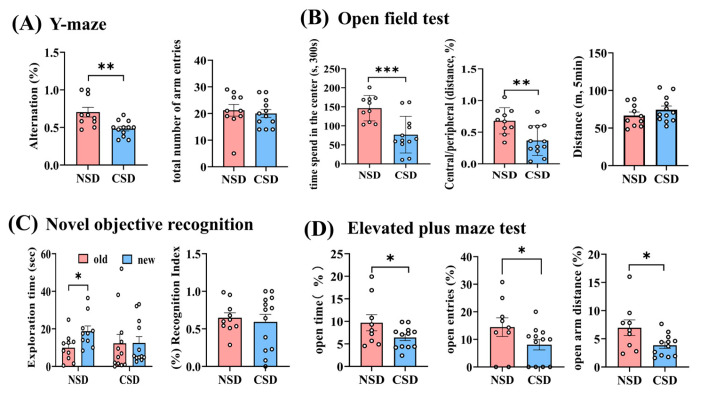
Behavioral characterization of mice after 6 months of chronic sleep deprivation. (**A**) Y-maze test; (**B**) open field test; (**C**) novel objective recognition test; (**D**) elevated plus maze test. All data were mean ± SEM, *n* = 10–12, * *p* ≤ 0.05, ** *p* ≤ 0.01, *** *p* ≤ 0.001, compared with NSD group; unpaired *t*-test analysis.

Utilizing the natural exploratory activity of rodents, a spontaneous alternation Y-maze test was employed to assess spatial learning memory capacity in CSD mice [21]. Both the total number of arm entries and the rate of spontaneous alternation were measured. The results revealed significantly lower alternation rates in CSD animals compared to non-sleep deprivation (NSD) mice (Figure 1A). This suggested cognitive–behavioral impairments in CSD mice. However, the total number of arm entries, indicative of locomotor ability, remained consistent across both groups, implying that locomotor capacity did not influence alternation behavior. New object recognition experiments were conducted to evaluate short-term memory capacity in sleep-deprived (SD) mice [22]. NSD mice demonstrated a stronger interest in the new object, spending significantly more time exploring it compared to the old one (Figure 1C). In contrast, no preference was observed in CSD mice, indicating impaired short-term memory. In the open field experiment, which assesses motor function and anxiety, no significant difference was observed in the total distance traveled between CSD and NSD mice (Figure 1B). However, significantly lower movement time and a decreased ratio of movement distance in the center to the periphery were exhibited by CSD mice, suggesting the development of anxiety-like behaviors. Furthermore, in the elevated plus maze test (Figure 1D), decreased frequency of entry into the open arm and reduced time spent moving within it were observed in CSD mice, indicating increased anxiety-like behavior. These findings suggested that CSD in mice led to cognitive–behavioral abnormalities resembling AD and anxiety-like moods.

### 2.2. Chronic Sleep Deprivation Induced Aβ Oligomerization and Synaptic Damage

**Figure 2:**
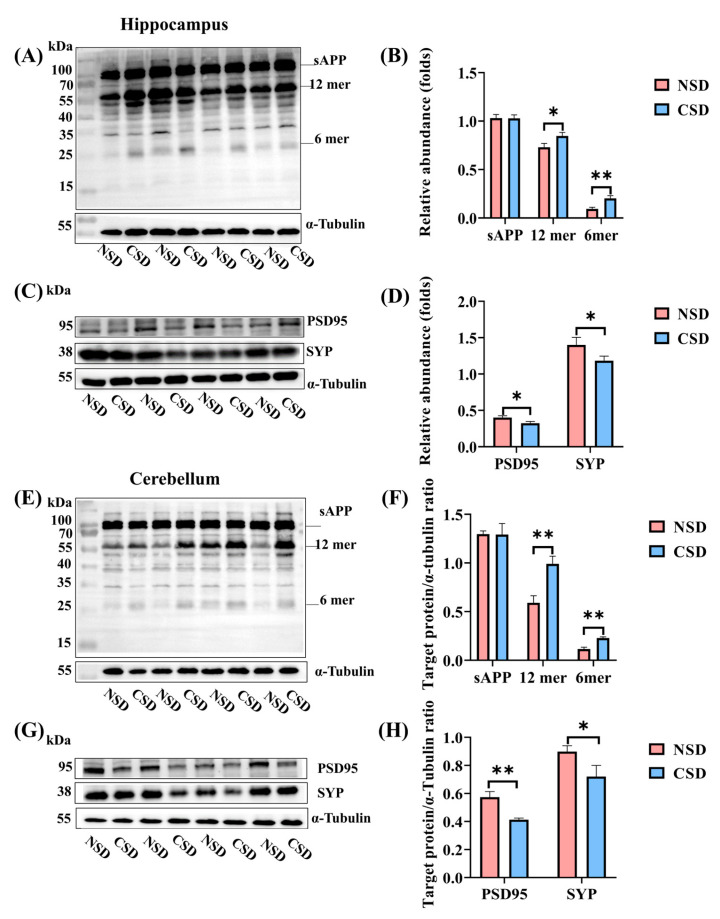
Effects of CSD on Aβ oligomerization and synaptic damage in the hippocampus and the cerebellum. Aβ oligomer expression in the hippocampus (**A**) and cerebellum (**E**) detected by Western blot (WB). Protein levels of PSD95 and SYP in the CSD and NSD groups in the hippocampus (**C**) and cerebellum (**G**) based on WB results. Bar graphs illustrate the protein expression levels of Aβ oligomers in the hippocampus (**B**) and cerebellum (**F**). Bar graphs illustrate the protein expression levels of PSD95 and SYP in the hippocampus (**D**) and cerebellum (**H**). All data are presented as mean ± SEM, * *p* ≤ 0.05, ** *p* ≤ 0.01, compared with the NSD group; unpaired *t*-test analysis.

To examine the pathological effects of CSD, we detected Aβ oligomers using a Western blot assay in mice after 6 months of CSD. Significant increases in Aβ oligomers, including hexamers (24 kDa) and dodecamers (56 kDa), were observed in both the hippocampus and cerebellum of CSD mice (Figure 2A,E), indicating that CSD induced the aggregation of Aβ oligomers in various regions of the mouse brain, with a particularly strong aggregation of Aβ dodecamers in the cerebellum. Given the heightened toxicity of Aβ dodecamers, the gene expression study was specifically conducted in the cerebellum [23]. Furthermore, CSD mice exhibited significant reductions in the expression of both synaptophysin (SYP) and postsynaptic density protein 95 (PSD95) in the hippocampus (Figure 2C,D). In the cerebellum, the expression of SYP and PSD95 was significantly reduced (Figure 2G,H), suggesting that 6 months of sleep deprivation resulted in synaptic damage in mice. Based on these findings, we hypothesized that Aβ oligomers and synaptic damage occur in the brains of mice subjected to CSD, leading to the development of AD-like cognitive abnormalities. Moreover, further exploration of gene expression changes in CSD mice using RNA-seq revealed insights into the mechanisms underlying cognitive dysfunction.

### 2.3. RNA Sequencing Unveiled the Scope of Gene Expression Alterations in the Cerebellum Triggered by Chronic Sleep Deprivation

**Figure 3:**
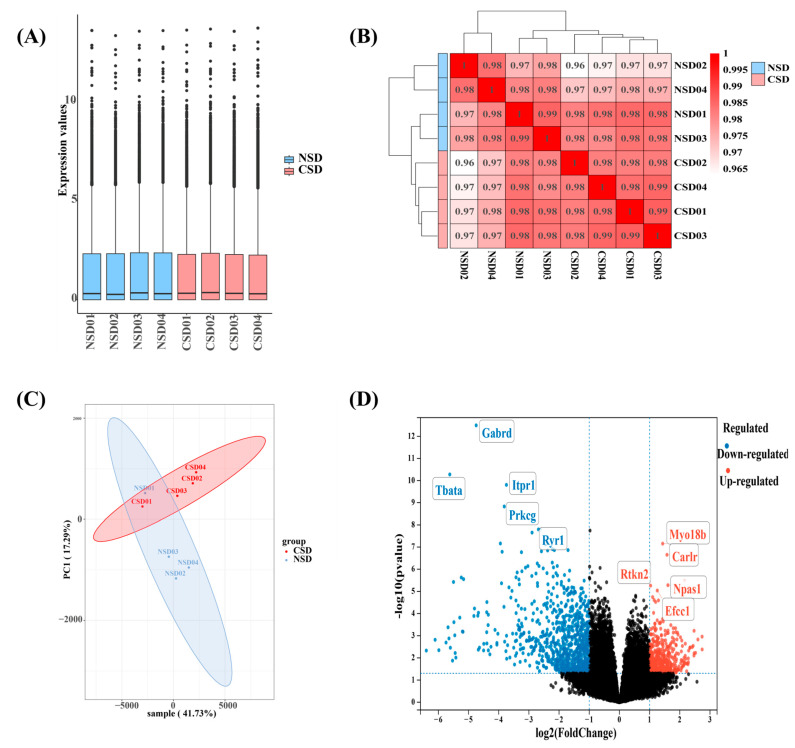
Results of RNA-seq data analysis after 6 months of sleep deprivation in the cerebellum. (**A**) Box plot displaying the level of gene expression in each sample. (**B**) Clustering heatmap based on correlation among samples. (**C**) Plots of principal component analysis (PCA) illustrating the relationship among eight samples. (**D**) Volcano plot showing differentially expressed genes meeting the criteria of *p*-value < 0.05 and fold change > 2.0. Red dots represent up-regulated genes in CSD vs. NSD, blue dots represent down-regulated genes in CSD vs. NSD, and black dots represent genes that were not differentially expressed in CSD vs. NSD.

Previous researchers have utilized microarray and RNA-seq technology to investigate the effects of acute sleep deprivation on gene expression in various brain regions [24,25]. However, the extent and specificity of gene regulation by CSD are still not fully understood. Therefore, we examined the impact of six months of CSD on gene expression changes in the cerebellum using RNA-seq technology. Gene expression levels were determined using featureCounts and corrected using the DESeq2 package. The uniformity of gene expression across samples is depicted in the box plot (Figure 3A), indicating the absence of sample outliers. Principal component analysis and distance matrix analysis heatmaps were employed to assess the RNA-seq data, demonstrating good clustering of biological replicates (Figure 3B,C), suggesting that the obtained data are suitable for further analysis. Following six months of sleep deprivation in wild-type mice, we identified 1317 genes that were significantly differentially expressed in the cerebellum, with fold change > 2.0 and *p*-value < 0.05. The volcano plot (Figure 3D) illustrated 475 significantly up-regulated genes, with the top five being Myo18b, Carlr, Npas1, Rtkn2, and Efcc1. Additionally, 842 genes were significantly down-regulated, with the top five being Gamma-aminobutyric acid type A receptor subunit Delta (Gabrd) (log2FC = −4.75, *p*-adj = 5.51 × 10^−9^), thymus, brain, and testes associated (Tbata) (log2FC = −5.62, *p*-adj = 4.58 × 10^−7^), inositol 1,4,5-trisphosphate receptor 1 (Itpr1) (log2FC = −3.74, *p*-adj = 9.01 × 10^−7^), protein kinase C, gamma (Prkcg) (log2FC = −3.81, *p*-adj = 6.45 × 10^−6^), and ryanodine receptor 1 (Ryr1) (log2FC = −2.68, *p*-adj = 4.41 × 10^−5^).

### 2.4. Genes Dysregulated by Chronic Sleep Deprivation Were Associated with Calcium Homeostasis and Synaptic Damage

**Figure 4:**
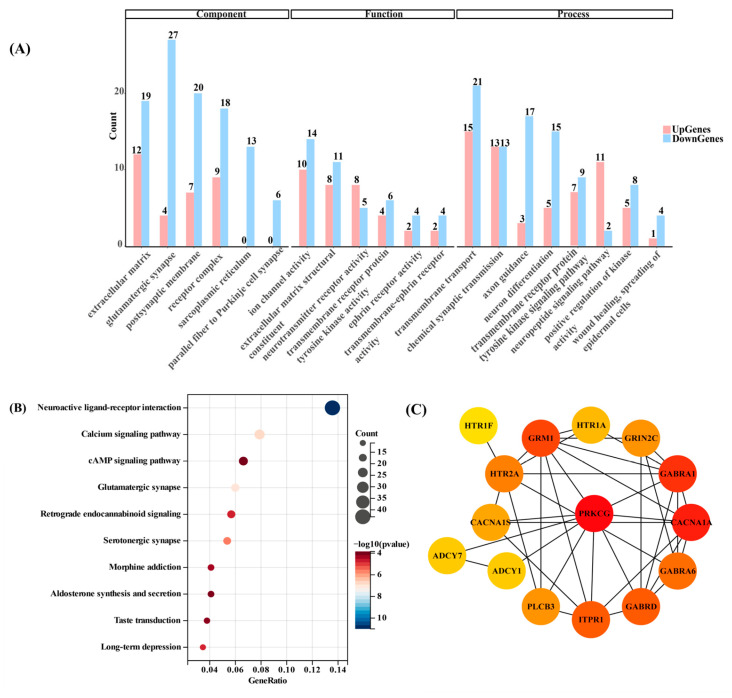
Functional analysis of DEGs after CSD. (**A**) GO enrichment analysis encompasses biological process, molecular function, and molecular composition in both downregulated and up-regulated genes after CSD. The top six GO terms with fold change > 2.0 and *p*-value < 0.05 are displayed. (**B**) KEGG enrichment analysis presents the top ten enriched pathways. (**C**) PPI network interactions of selected hub genes.

To explore the regulatory function of CSD on cognitive impairment in mice, gene ontology (GO) enrichment analysis of DEGs in the cerebellar regions was conducted. A total of 2672 GO entries were enriched, with the top 6 entries based on DEGs categorized into biological process (BP), molecular function (MF), and cellular component (CC) (Figure 4A). GO functional analysis identified 1850 BP terms, 499 MF terms, and 323 CC terms (Table S1). Notably, terms such as “transmembrane transport”, “chemical synaptic transmission”, and “neuron differentiation” were among the most significantly enriched BP entries, while “extracellular matrix”, “glutamatergic synapse”, and “postsynaptic membrane” were notable CC entries. In terms of MF, “ion channel activity” and “neurotransmitter receptor activity” were highly enriched. Furthermore, KEGG pathway enrichment analysis using the clusterProfiler package revealed significant enrichment of DEGs in 274 typical pathways. The top ten pathways with the highest abundance of DEGs, including “neuroactive ligand–receptor interaction”, “calcium signaling pathway”, and “glutamatergic synapse”, were visualized in dot diagrams (Figure 4B). Specifically, 29 related DEGs were identified in the calcium signaling pathway, with 11 up-regulated and 18 down-regulated genes, while 19 genes were affected in the glutamatergic synapse pathway.

Moreover, overlapping DEGs were observed across multiple pathways. For instance, Grin2c, Htr4, and Htr6 were expressed in all four pathways, while Itpr1, Prkcg, and others were expressed in three pathways. To further analyze these overlapping DEGs, a protein–protein interaction (PPI) network was constructed using Cytoscape. The enriched route overlapping DEGs were used as seeds for establishing the PPI network linked to the development of cognition and memory during sleep deprivation. Genes are represented as nodes in a PPI network, while interactions are represented as edges. The degree of interaction between the genes in question increases with the number of wires and the node’s darkness. The Prkcg gene nodes were the darkest and had the greatest interactions with other genes (Figure 4C). Furthermore, the expression of the Prkcg gene dropped by 14-fold in the CSD group compared to the NSD group following sleep deprivation. Consequently, Prkcg was identified as a key gene and subjected to further investigation and verification of its expression in relation to other relevant genes.

### 2.5. Validation of Gene Expression Changes in the Cerebellum after Sleep Deprivation

**Figure 5:**
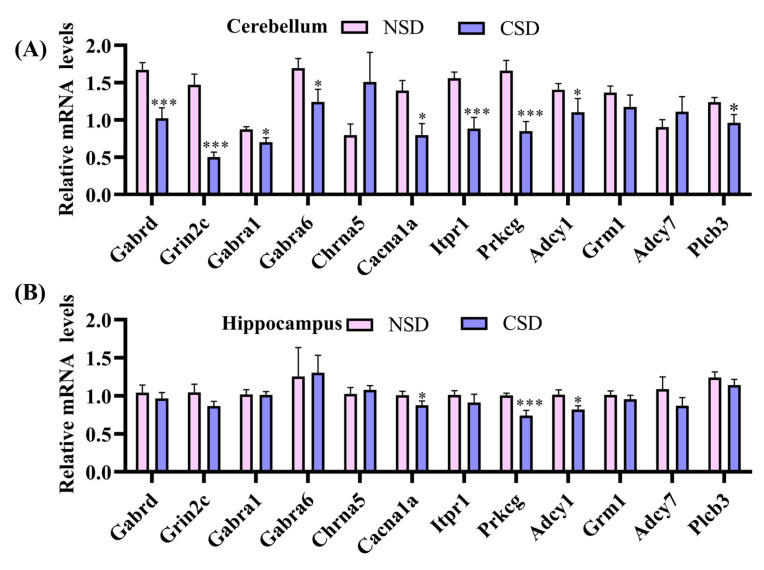
Validation of mRNA expression of 12 hub genes screened in cerebellum (**A**) and hippocampus (**B**). (* *p* ≤ 0.05, *** *p* ≤ 0.001, compared with the NSD group; unpaired *t*-test analysis).

Based on functional and pathway enrichment analyses of DEGs, independent sleep deprivation experiments were conducted to validate the findings from RNA-seq. Specifically, 12 genes involved in regulating calcium homeostasis, synaptic damage, and neuroactive ligand–receptor interactions—Gabrd, Grin2c, Gabra1, Gabra6, Chrna5, Cacna1a, Itpr1, Prkcg, Grm1, Adcy1, Adcy7, and Plcb3—were selected for RT-qPCR analysis. There was a significant down-regulation of mRNA expression for Gabrd, Grin2c, Gabra1, Gabra6, Cacna1a, Itpr1, Prkcg, and Plcb3 in the cerebellar region of CSD mice compared to the NSD group (Figure 5A). Notably, Gabrd, Grin2c, Itpr1, and Prkcg exhibited particularly pronounced decreases in expression. Conversely, the expression of Chrna5 and Adcy7 showed a tendency to increase, consistent with the sequencing results.

Previous studies have reported the vulnerability of the hippocampus to acute sleep deprivation, characterized by alterations in gene expression, cell signaling, and protein synthesis [23]. However, there have been few reports on the impact of CSD on the hippocampus. Therefore, we examined the mRNA expression of certain genes related to calcium homeostasis and synaptic damage in the hippocampus of CSD mice. The mRNA expression of Cacna1a and Prkcg was significantly down-regulated (Figure 5B). Remarkably, these two genes showed substantial alterations in the same direction in both the cerebellum and hippocampus following CSD in mice, consistent with the RNA-seq data. This suggested that disturbances in calcium signaling homeostasis may contribute to the cognitive memory impairment induced by CSD.

**Figure 6:**
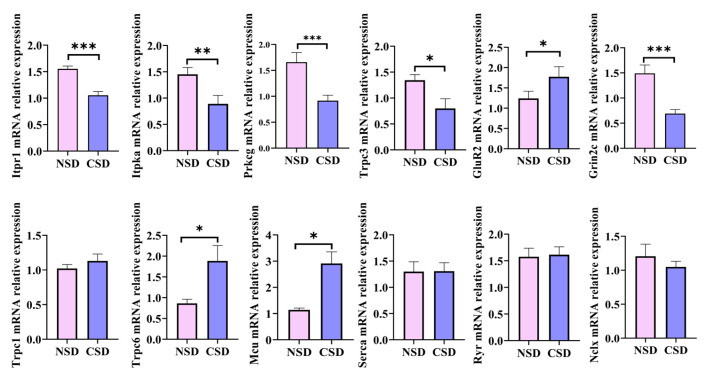
RT-qPCR validation of calcium signaling-related gene expression. (* *p* ≤ 0.05, ** *p* ≤ 0.01, *** *p* ≤ 0.001, compared with the NSD group; Unpaired *t*-test analysis).

Subsequently, the mRNA expression of genes associated with calcium signaling was assessed using RT-qPCR. CSD mice exhibited significant decreases in the mRNA expression of Itpr1, the gene regulating the endoplasmic reticulum (ER) calcium efflux protein IP3R1. However, the mRNA expression of RYR, the ER calcium efflux channel, and SERCA, the ER Ca^2+^-ATPase, showed no significant differences following six months of CSD (Figure 6). Interestingly, there was a notable down-regulation in the cytoplasmic expressions of Itpka and Prkcg mRNA, which was involved in controlling calcium channel feedback. While TRPC1 expression remained constant, TRPC3 expression showed a dramatic decrease, and TRPC6 expression significantly increased. Additionally, the mRNA expression of the mitochondrial unidirectional calcium transporter (MCU) was markedly down-regulated, and the mRNA expression of the Na^+^/Ca^2+^ exchangers remained constant. Moreover, the mRNA expression of Grin2c, a subunit composition of the calcium intracellular channel receptor, NMDAR, was drastically reduced in CSD mice. These findings suggested a dysregulation of calcium signaling in the cerebellum associated with CSD.

### 2.6. Chronic Sleep Deprivation Impaired the Synthesis of Memory-Related Proteins in the Cerebellum

**Figure 7:**
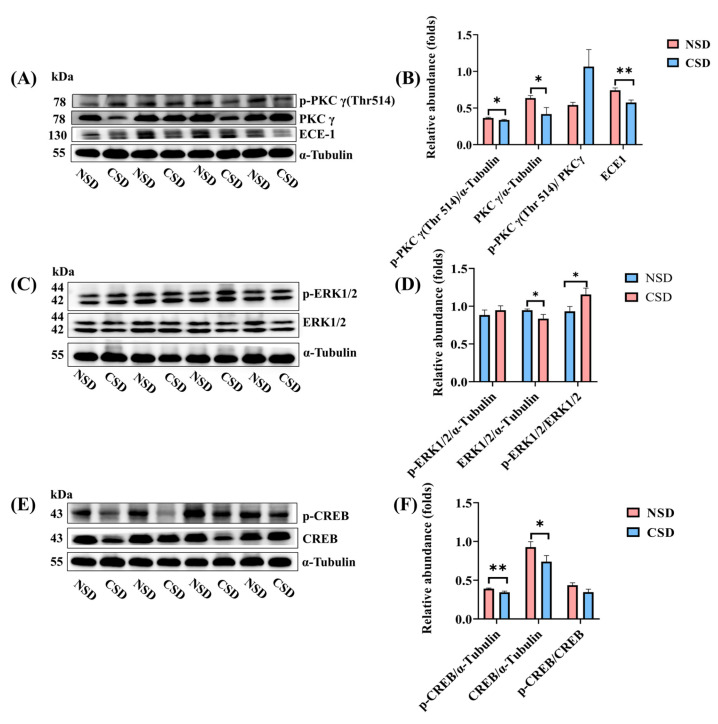
Chronic sleep deprivation affected the PKCγ/ERK/CREB signaling pathway in the cerebellum. (**A**,**B**) Representative Western blots and quantification of phosphorylated PKCγ, total PKCγ, ECE1, and their ratio in cerebellar extracts from NSD and CSD mice. (**C**,**D**) Representative Western blots and quantification of phosphorylated ERK, total ERK, and their ratio in cerebellar extracts from NSD and CSD mice. (**E**,**F**) Representative Western blots and quantification of phosphorylated CREB, total CREB, and their ratio in cerebellar extracts from NSD and CSD mice. All data are presented as mean ± SEM, * *p* ≤ 0.05, ** *p* ≤ 0.01, compared with the NSD group; unpaired *t*-test analysis.

Using RNA-seq data and mRNA validation, Western blot analysis was employed to characterize the protein level of Prkcg. The data (Figure 7A,B) demonstrated a significant decrease in PKCγ expression and phosphorylation levels at the Thr514 location in CSD mice. Additionally, there was a notable reduction in the protein expression of endothelin-converting enzyme (ECE1) in CSD mice. ECE1 plays a crucial role in degrading Aβ proteins, and its decreased expression inhibits Aβ degradation, leading to Aβ accumulation in the brain [26,27]. Consequently, we hypothesized that CSD-induced reduction in PKCγ expression, down-regulation of ECE1 expression, dysregulation of Aβ degradation, and subsequent Aβ accumulation in the brain contribute to impaired learning and memory function in mice.

Furthermore, the phosphorylation level of extracellular regulated protein kinases (ERK 1/2) at site 42/44 was significantly increased, while the phosphorylation level of cAMP-response element binding protein (CREB) was decreased in CSD mice (Figure 7D,F). CREB is known to play a crucial role in neuronal plasticity and long-term memory formation, and its downregulation is associated with impaired spatial memory formation [28,29]. Moreover, reduced CREB activity has been linked to deficits in hippocampal long-term potentiation (LTP) [30]. Given that CREB downregulation is implicated in the pathophysiology of AD, increasing CREB expression has been proposed as a potential therapeutic target [31,32,33].

Finally, the expression of postsynaptic density protein 95 (PSD95) and synaptophysin (SYP) was significantly decreased in CSD mice according to Western blot analysis (Figure 2D,H). These results collectively suggested that CSD down-regulated the expression of memory-associated protein kinase PKCγ, leading to over-activation of ERK, significant reduction in CREB expression, and subsequent impairment of memory and learning function in CSD mice.

## 3. Discussion

The majority of studies conducted on AD have been on determining how harmful Aβ is to the hippocampus’s structure; however, as AD patients can also have problems with speech, vision, or motor function, it is possible that Aβ pathology transcends the hippocampus [34]. Research on both familial (FAD) and sAD cases has demonstrated that AD patients’ cerebellums are vulnerable [15]. The cerebellar cortex of AD patients showed higher concentrations of Aβ oligomers [35]. In addition, cases of early-onset FAD resulting from mutations in the gamma-secretase catalytic subunit (PSEN1) exhibit high levels of hyperphosphorylated tau protein, cerebellar Aβ accumulation, and loss of Purkinje cells (PC) [14,18]. The precise function of the cerebellum in cognitive processes has emerged as a highly intriguing topic.

Previous studies have predominantly focused on the effects of acute sleep deprivation on cognitive dysfunction, leaving the extent and specificity of gene regulation by CSD incompletely understood [2,36,37]. In the present study, we investigated gene and molecular expression changes associated with memory and cognitive impairment following six months of CSD in mice cerebellum and hippocampus. After six months of sleep deprivation, CSD mice exhibited behavioral deficits, including reduced spatial memory capacity, diminished short-term learning memory capacity, and anxiety-like mood compared to the NSD group. Subsequently, we characterized Aβ deposition in the brains of both groups of mice using Western blot analysis. Our experimental results revealed a significant increase in Aβ oligomers hexamer and dodecamer in the brains of CSD mice, indicating that sleep deprivation exacerbated Aβ deposition. Additionally, the significant decrease in the expression of PSD95 and SYP suggested synaptic damage in the brains of CSD mice. To elucidate the mechanisms underlying Aβ oligomerization and synaptic damage induced by CSD, we conducted RNA-seq to identify significantly differentially expressed genes following CSD and validated their expression analysis.

In the RNA-seq analysis of mouse cerebellar samples, a total of 1317 differential genes were identified, comprising 475 significantly up-regulated genes and 842 significantly down-regulated genes. GO enrichment and KEGG enrichment analyses revealed calcium homeostasis and synaptic damage as pivotal events in the CSD model. Intersection analysis of differential genes enriched from the GO and KEGG pathways identified 12 hub genes as significant differentially expressed functional genes for CSD, including Gabrd, Grin2c, Gabra1, Gabra6, Chrna5, Cacna1a, Itpr1, Prkcg, Adcy1, Grm1, Adcy7, and Plcb3. Verification through RT-qPCR experiments confirmed the consistency with RNA-seq results. Furthermore, the mRNA expression of 12 genes related to calcium homeostasis, including Itpr1, Itpka, Prkcg, Trpc3, Trpc1, Trpc6, GluR2, Grin2c, Mcu, Serca, Ryr, and Nclx, was characterized based on enrichment analysis. Among them, the mRNA expression of Itpr1, Itpka, Prkcg, Grin2c, and Trpc3 was significantly reduced, while the mRNA expression of Serca and Ryr remained largely unchanged. Impairment of intracellular Ca^2+^ homeostasis is considered to be a key pathological factor leading to age-related neuronal dysfunction [38]. Intracellular Ca^2+^ concentration is regulated by ion channels in the cytoplasmic membrane and endoplasmic reticulum (ER), mitochondrial membranes, and certain intracellular calcium-binding proteins.

In this study, the Cacna1a gene, encoding a pore-forming protein for voltage-gated calcium channels, was detected to have significantly reduced mRNA expression in both the cerebellum and hippocampus, but WB protein assays showed no difference (Figure S1). We speculated that it may be the slower rate of degradation of its proteins. Grin2c encodes the n-methyl-d-aspartate (NMDA) receptor subunit, the activation of which causes calcium influx. Reduced expression of Grin2c mRNA may lead to changes in the composition of NMDAR subunits, inability to properly activate NMDAR, and reduced influx of extracellular calcium ions. The AMPA receptor is one of the pro-ionotropic glutamate receptors, and its permeability to Ca^2+^ and other divalent ions is determined by the amount of its GluR2 subunit. When the AMPA receptor contains the GluR2 subunit, the permeability to calcium ions is extremely low, so it is called a calcium-impermeable AMPA receptor [39]. Significantly increased levels of GluR2 mRNA expression indicated that the AMPA was a calcium-impermeable receptor, which restricted the influx of extracellular calcium ions into the intracellular compartment. It is worth noting that the expression of the ER efflux channel IP3R1 was significantly reduced, while the expression of Serca and Ryr remained largely unchanged. Reduced expression of IP3RI leads to reduced binding to IP3 and reduced calcium ion efflux via IP3R. TRPC3 channel-mediated calcium influx is reduced, and selective uptake of calcium ions by mitochondrial unidirectional transport proteins is increased, limiting intracytoplasmic calcium ion concentration. The low intracytoplasmic calcium ion concentration resulted in the failure of PKCγ activation by binding to DAG, and the WB assay showed a significant decrease in PKCγ expression.

PKC plays a significant role in numerous forms of learning and memory. Mice lacking PKC-γ exhibit cognitive deficits and problems in hippocampus synaptic plasticity, indicating that PKC-γ is particularly relevant to learning and memory among the several PKC isoforms [40]. PKCγ-deficient mice exhibit altered hippocampal long-term potentiation (LTP), mild deficits in spatial and contextual learning, and impaired motor coordination due to sustained multiple innervations of Purkinje cells by climbing fibers [41,42]. In contrast, it has been demonstrated that overexpressing PKCγ in the hippocampal CA1 area helps prenatal stress rats regain their capacity for spatial learning and memory [43]. High expression of PKCγ in hippocampal pyramidal cells and cerebellar Purkinje cells has been linked to the regulation of synaptic plasticity, including LTP and LTD [44,45]. In RNA-seq analysis, the CSD group exhibited a 14-fold differential fold change in Prkcg (PKCγ) compared to the NSD group. Additionally, in cerebellar RT-qPCR validation, Prkcg displayed the most significant differential expression after CSD (*p* = 0.0008). A notable decrease in mRNA expression of Prkcg was also observed in the hippocampus (*p* = 0.0009). A significant decrease in PKCγ expression was also detected at the protein level in CSD mice, with a notably lower level of phosphorylation at the Thr514 site. It has been reported that increased neuronal PKC activity enhances Aβ clearance and reduces AD neuropathology by elevating endothelin-converting enzyme activity [27]. This study also observed a significant decrease in ECE1 expression in the CSD group of mice. Consequently, we hypothesized that CSD diminishes PKCγ expression, thereby regulating the decrease in ECE1 expression, leading to reduced Aβ clearance and deposition of Aβ oligomers. This cascade may contribute to AD-like memory and cognitive abnormalities in CSD mice, suggesting PKCγ as a potential novel target for the pharmacological treatment of AD.

Extracellular signal-regulated kinase (ERK) plays a crucial role in regulating cell growth, differentiation, apoptosis, and the cellular response to stress. Long-term memory formation necessitates activation of the ERK-CREB signaling pathway [31,46,47]. Feng suggested that PKC isozymes mediate the phosphorylation of ERK and CREB [48]. ERK phosphorylation significantly increased, while CREB expression and phosphorylation markedly decreased (Figure 7F). Hence, we proposed that the reduced expression of PKCγ in CSD mice triggered hyperphosphorylation of ERK, thereby negatively regulating CREB, leading to reduced expression of memory-related proteins in the brain. This cascade may contribute to the development of memory dysfunction in CSD mice.

PKCγ serves as a crucial protease regulating Aβ degradation and also modulates the ERK/CREB pathway, thereby influencing the synthesis of memory-related proteins. Consequently, regulating the expression of PKCγ may offer a potential avenue to alleviate cognitive memory dysfunction associated with CSD, presenting novel targets and ideas for the development of drugs targeting sporadic AD. Subsequently, further experiments will be conducted to confirm the regulatory role of PKCγ on the downstream ERK/CREB pathway.

## 4. Materials and Methods

### 4.1. Animal Care and Sleep Deprivation

The study was conducted at the Animal Testing Center of China Pharmaceutical University (Nanjing, China) in accordance with the relevant institutional and national guidelines for the care and use of laboratory animals. Ten-month-old female C57BL/6 mice were provided by Bikai Experimental Animal Co., Ltd., Shanghai, China. Mice were housed in temperature- and humidity-controlled (24 ± 2 °C and 55 ± 5% humidity, respectively) rooms, with a 12 h light/dark cycle and free access to food and water. After one week of acclimatization feeding, the mice were divided into two groups (n = 12): non-sleep deprivation (NSD) and chronic sleep deprivation (CSD) groups. Sleep deprivation in mice was performed by ZL-013 sleep deprivation drums purchased from Anhui Yaokun Biotechnology Co., LTD., Fuyang, China. The setup included a plexiglass cylinder (400 × 390 mm), a revolving rod (390 mm), a main box with display (410 × 410 × 100 mm), water bottles, food containers, and light sources. The revolving rod disrupted the mice’s sleep for six months, operating at a speed of 5 rpm/min from 8:00 AM to 6:00 PM. The entire process was conducted under light conditions, with the rod’s direction randomly alternated. The NSD mice had a rotating speed of zero and were housed in the same cylinder. All animals had unrestricted access to food and water. At the end of sleep deprivation, the sixteen-month-old mice were sacrificed, and brain tissue was sectioned for subsequent experiments.

### 4.2. Y-Maze Test

The Y-maze test primarily leverages the natural tendencies of rodents to explore new and diverse environments, effectively assessing their spatial working memory. This is because mice must recall previously explored directions each time they change their exploration path [21]. In this test, mice are placed at the end of any arm of the Y-maze and allowed to freely explore for 8 min while their behavioral patterns are recorded by a camera system. Subsequently, the total number of arm entries and alternations are documented. “Total number of entries” refers to instances where mice fully enter an arm of the maze, with all four feet inside, while “alternation” denotes the consecutive sequence of arm entries. After each mouse completes the trial, it is returned to its cage, and any residual odor inside the Y-maze arms is eliminated using 75% alcohol before the next experiment. The spontaneous alternation rate is calculated as follows:

Alternation (%) = total number of alternations/(total number of arm entries − 2) × 100(1)

### 4.3. Novel Objective Recognition Test

The novel object recognition (NOR) test assesses the memory capacity of the subjects by observing the duration they spend investigating familiar and novel objects [49]. In Phase 1, mice freely roamed within the experimental device without any objects for 10 min. Subsequently, in the second stage, the training stage, two identical objects (labeled A and B) were positioned in the device, each 10 cm away from the adjacent walls. The mice were then introduced into the setup with their backs turned to the objects, equally spaced from each, while a camera and software system recorded their exploration time on each object. The number of instances the animals interacted with each object was tallied within a 5 min interval. During the testing phase, two hours after the completion of the training stage—serving as the memory retention interval—one of the identical objects was substituted with a novel one (labeled as object C). Mice were placed back into the device with their backs facing the objects, equally spaced from each, for a 5 min session. Before commencing each subsequent mouse trial, the experimental device underwent thorough cleaning with alcohol to eliminate any residual odors and ensure the absence of mouse-related scents or waste. The duration spent by the mice investigating object A was denoted as F, while the exploration time for the new object C was labeled as N. The recognition index (RI), representing the ratio of time spent exploring the new object to the total exploration time, was calculated using the following formula:

RI% = N/(N + F) × 100(2)

### 4.4. Open Field Test

The open field test (OFT) detects spontaneous activity and exploratory behavior in rats or mice. Animals tend to exhibit fear towards new and open environments, leading them to frequently occupy the peripheral area and avoid the central area. However, their exploratory behavior may encourage movement towards the central area and, consequently, the manifestation of anxiety symptoms [50]. The bottom of the mouse field experiment (50 × 50 × 40 cm) box was divided into the central area and the peripheral area. At the beginning of the experiment, the mice were quickly placed in the central area of the experimental box, and the software was immediately opened to record the activities of the mice in the box for a period of 5 min. The total distance of the horizontal movement and the residence time in the central area were recorded using the Digbehv software (version 2.0; Shanghai Jiliang Software Technology Co., Ltd., Shanghai, China).

### 4.5. Elevated Plus Maze Test

The elevated plus maze (EPM) test is utilized to measure the anxiety levels of animals by exploiting the contradictory behaviors between the animal’s exploratory characteristics towards new surroundings and its discomfort with the raised open arms [51]. The EPM comprises a central square measuring 5 × 5 cm and four arms of identical size measuring 45 × 5 × 15 cm. These arms protrude from the central platform, forming two exposed and two enclosed arms. The device is positioned approximately 50 cm above the ground. The mice were introduced into the maze via the central compartment, with their initial orientation toward the closed arms. They were then observed for a timeframe of 5 min. The recorded observations included the number of entries, duration of time, and the distance traveled in the open arms. After completing the experiment, the mice were removed, and both arms were cleaned and sprayed with alcohol to eliminate odors. The data were analyzed using Digbehv software.

### 4.6. RNA Extraction

Total RNA was extracted from brain tissue using the TRIzol reagent, according to Chomczynski et al. [52]. The concentration and purity of the extracted RNA were detected using the NanoPhotometer^®^ spectrophotometer (IMPLEN, Westlake Village, CA, USA). RNA integrity was determined by agarose gel electrophoresis, and RIN value was determined by RNA Nano 6000 Assay Kit of the Bioanalyzer 2100 system (Agilent Technologies, Santa Clara, CA, USA).

### 4.7. RNA Library Preparation and Sequencing

For the purpose of building libraries, total cerebellum RNA greater than 1 ug was used as input material. The sequencing library was constructed using Illumina’s NEBNext^®^ UltraTM RNA Library Prep Kit (NEB, Ipswich, MA, USA). The library fragments were purified using the AMPure XP technology (Beckman Coulter, Beverly, MA, USA) in order to identify cDNA fragments that were predominantly 250–300 bp in length. After the library was constructed, preliminary quantification was carried out using the Qubit2.0 Fluorometer. The library was diluted to 1.5 ng/μL, and then the insert size of the library was detected by the Agilent 2100 bioanalyzer. After the insert size met expectations, the effective concentration of the library was precisely measured using RT-qPCR.

### 4.8. RNA Sequencing Analysis

After passing the library check, 150 bp paired-end reads were generated by pooling several libraries based on the effective concentration and target downstream data volume needed for Illumina sequencing. The sequencer captures fluorescence signals and converts them into sequencing peaks using computer software, thereby obtaining sequence information for the fragments being tested. The initial raw data in fastq format was processed using in-house perl scripts to obtain clean data by removing reads containing adapters, poly-N, and low-quality reads. Additionally, the clean data’s Q20, Q30, and GC content was calculated. All subsequent analyses were performed on high-quality, clean data. The reference genome and gene model annotation files were directly downloaded from the genome website. The index of the reference genome was constructed using Hisat2 v2.0.5. The paired-end clean reads were then aligned to the reference genome using Hisat2 v2.0.5. The differentially expressed genes (DEGs) were identified based on their multiplicity and significance level of differences. DEGs were identified using the DESeq2 R package (version 1.16.1). Fold change > 2.0 and *p* < 0.05 were considered as significant differentially expressed genes.

### 4.9. Bioinformatics Analysis

The clusterProfiler R package v4.0 was used to conduct Gene Ontology (GO) enrichment analysis of DEGs, correcting for gene length bias. GO terms with corrected *p*-values less than 0.05 were considered significantly enriched by DEGs. Statistical enrichment of DEGs in the Kyoto Encyclopedia of Genes and Genomes (KEGG) pathway was analyzed using Sangerbox 3.0 data analysis platform. Protein–protein interaction (PPI) analysis was conducted on differentially expressed genes using the STRING database, which includes both known and predicted protein interactions.

### 4.10. Quantitative Real-Time PCR (RT-qPCR)

One microgram of RNA was reverse transcribed into cDNA using the Reverse Transcription Kit (Vazyme, R323). The qPCR experiments were conducted with gene-specific primers using the ChamQ SYBR qPCR Master Mix (Without ROX) (Vazyme, Q321) on a LightCycler 96 (Roche, Pleasanton, CA, USA). Relative quantification of target genes was performed using GAPDH as the housekeeping gene. Table 1 contains a list of primers utilized in this investigation.

### 4.11. Western Blot

The partitioned brain tissues were lysed using RIFA, and the protein concentration was determined using a BCA kit (Biyun Tian Co., Shanghai, China). Equal amounts of protein samples were separated by 8–12% SDS-PAGE electrophoresis, followed by transfer to PVDF membranes. The membranes were then blocked with 5% milk powder, incubated with primary antibodies overnight, followed by corresponding secondary antibodies at room temperature for 1 h, and finally detected using an enhanced chemiluminescence substrate. The intensity of the bands was quantified using ImageJ software (Version 1.53e).

### 4.12. Statistical Analyses

Statistical analysis of this research was conducted using an unpaired Student’s *t*-test in GraphPad Prism (v. 9.0). The results were presented as mean ± SEM, with statistical significance defined as *p* < 0.05.

## 5. Conclusions

In this study, we established a CSD mouse model and conducted RNA-seq analysis and validation at both the gene and protein levels, uncovering that CSD impaired spatial working memory and induced Aβ oligomerization and synaptic damage in mice. RNA sequencing unveiled the genes dysregulated by CSD were associated with calcium homeostasis and synaptic damage. CSD down-regulated PKCγ expression, decreased ECE1 expression, impaired Aβ degradation, and affected the PKCγ/ERK/CREB pathway and the synthesis of PSD95 and SYP proteins. Regulating the expression of PKCγ may offer a potential avenue to alleviate cognitive memory dysfunction associated with CSD, presenting novel targets and ideas for the development of drugs targeting sporadic AD.

## Figures and Tables

**Table 1:** Primers for RT-qPCR analysis.

Target	Forward (5′-3′)	Reverse (5′-3′)
Gabrd	ATTGGGGACTACGTGGGCT	CAAGCGCCACATTCACAGG
Grin2c	GGGATCTGCCATAACGAGAAG	GCACTGAGTGTCGAAGTTTCCA
Gabra1	AAAAGTCGGGGTCTCTCTGAC	CAGTCGGTCCAAAATTCTTGTGA
Gabra6	TGCCCAAGCTCAACTTGAAGA	GCCGTAGACGGTTGTCATAGC
Chrna5	ATGGCACTGTCACTTGGACG	GCCGAATTTCATGGAGCAATTTT
Cacna1a	GGACAGGTAGGTCCGAGGT	CTCTGCTGTGTAGTCGCAGT
Itpr1	CGTTTTGAGTTTGAAGGCGTTT	CGTTTTGAGTTTGAAGGCGTTT
Prkcg	CTCATTCCTATGACCCC	ATCCCAATCCCACACCTC
Adcy1	GTCACCTTCGTGTCCTATGCC	TTCACACCAAAGAAGAGCAGG
Grm1	TGGAACAGAGCATTGAGTTCATC	CAATAGGCTTCTTAGTCCTGCC
Adcy7	AAGGGGCGCTACTTCCTAAAT	GTGTCTGCGGAGATCCTCA
Plcb3	CGCGGGAGTAAGTTCATCAAA	CCTCCATGTTGGGTCCTGTC
Itpka	GACTCGGAGGACGATCTGCT	CTTCTGCCAGTGGCTTTTCTG
Trpc3	GCGAGCAAGAACTGCGAGAT	TGCACCACCTCGTACTTATGG
Glur2	TTCTCCTGTTTTATGGGGACTGA	CTACCCGAAATGCACTGTATTCT
Trpc1	GTCGCACCTGTTATTTTAGCTGC	TGGGCAAAGACACATCCTGC
Trpc6	AGCCAGGACTATTTGCTGATGG	AACCTTCTTCCCTTCTCACGA
Mcu	CGCCGTTTCCAGTTGAGAGA	TACTGCAATATGCGGCTCCC
Serca	AAAGACTCCTGGCCCTGACTA	CGATGATGCAGCCTACAATCC
Ryr	CGCACACAGTCGTATGTACCT	TAATCCCACGTCAAAGGCCAA
Nclx	CTCAGCTCTGCTACTCAACCA	CACGAGGTCCATCCTCATCC

## Data Availability

Data are contained within the article and Supplementary Materials.

## Supplementary Materials

The following supporting information can be downloaded at: https://www.mdpi.com/article/10.3390/ijms252111634/s1.
