# Supplementary material for: Chronic Sleep Deprivation Altered the Expression of Memory-Related Genes and Caused Cognitive Memory Dysfunction in Mice
Source: Int J Mol Sci. 2024 Oct 29;25(21):11634. doi: 10.3390/ijms252111634 (PMC11546330; doi:10.3390/ijms252111634)
Supplement: Supplementary file 1 [file ijms-25-11634-s001.zip › ijms-3184034-supplementary.pdf]

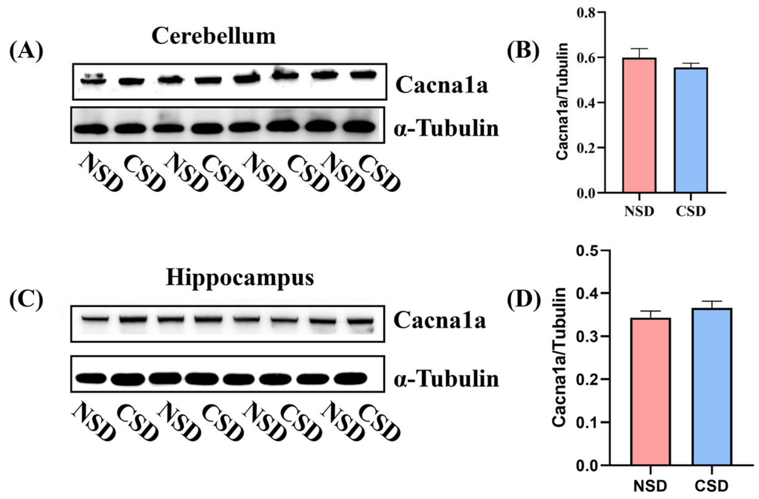

Figure S1. Effects of CSD on the expression of Cacna1a in the hippocampus and the cerebellum. The expression of Cacna1a in the cerebellum (**A**,**B**) and hippocampus (**C**,**D**).

Table S1. Gene ontology (GO) enrichment analysis.
